# Trends in the Medical Management of Patients With Heart Failure

**DOI:** 10.4021/jocmr1376w

**Published:** 2013-04-23

**Authors:** Samuel W. Joffe, Matthew DeWolf, Jeffrey Shih, David D. McManus, Frederick A. Spencer, Darleen Lessard, Joel M. Gore, Robert J. Goldberg

**Affiliations:** a Department of Medicine, University of Massachusetts Medical School, Worcester, MA, USA; b Department of Quantitative Health Sciences, University of Massachusetts Medical School, Worcester, MA, USA; c Department of Medicine, McMaster University Medical Center, Hamilton, Ontario, Canada

**Keywords:** Acute heart failure, Time trends, Population surveillance

## Abstract

**Background:**

Despite the availability of effective therapies, heart failure (HF) remains a highly prevalent disease and the leading cause of hospitalizations in the U.S. Few data are available, however, describing changing trends in the use of various cardiac medications to treat patients with HF and factors associated with treatment. The objectives of this population-based study were to examine decade-long trends (1995 - 2004) in the use of several cardiac medications in patients hospitalized with acute decompensated heart failure (ADHF) and factors associated with evidence-based treatment.

**Methods:**

We reviewed the medical records of 9,748 residents of the Worcester, MA, metropolitan area who were hospitalized with ADHF at all 11 central Massachusetts medical centers in 1995, 2000, 2002, and 2004.

**Results:**

Between 1995 and 2004, respectively, the prescription upon hospital discharge of beta-blockers (23%; 67%), angiotensin pathway inhibitors (47%; 55%), statins (5%; 43%), and aspirin (35%; 51%) increased markedly, while the use of digoxin (51%; 29%), nitrates (46%; 24%), and calcium channel blockers (33%; 22%) declined significantly; nearly all patients received diuretics. Patients in the earliest study year, those with a history of obstructive pulmonary disease or anemia, incident HF, non-specific symptoms, and women were less likely to receive beta blockers and angiotensin pathway inhibitors than respective comparison groups. In 2004, 82% of patients were discharged on at least one of these recommended agents; however, only 41% were discharged on medications from both recommended classes.

**Conclusions:**

Our data suggest that opportunities exist to further improve the use of HF therapeutics.

## Introduction

Heart failure is a highly prevalent, morbid, and costly disease, affecting nearly 6 million Americans and causing more than 275,000 deaths annually [1]. Heart failure (HF) is also the leading cause of hospital admissions in the U.S.

Over the past 2 decades, numerous randomized controlled trials have demonstrated improved survival in patients with HF with reduced ejection fraction (HFrEF) treated with beta blockers, angiotensin converting enzyme (ACE) inhibitors, and angiotensin receptor blockers (ARB’s) [2-8]. In certain populations, aldosterone antagonists and the combination of nitrates plus hydralazine have demonstrated important health benefits [9, 10]. Clinical trials have shown a lack of benefit on long-term survival in patients with HF treated with non-dihydropyridine calcium channel blockers or digoxin [11, 12], though the DIG study showed fewer re-hospitalizations and enhanced patient exercise capacity with digoxin [11]. Although diuretics are a therapeutic mainstay in patients with HF, no large randomized controlled trial has been conducted demonstrating a survival benefit from the use of these agents. Aspirin and statins have been shown to increase survival in patients with coronary artery disease (with or without accompanying HF), but not in patients with non-ischemic HF [13].

The results of these trials and other evidence have been incorporated into the ACC/AHA Clinical Practice Guidelines for Congestive Heart Failure, which were first published in 1995, with subsequent updates [14-17]. For patients with HF with reduced ejection fraction (HFrEF), beta blockers, ACE inhibitors, and ARB’s receive a Class I, level of evidence A recommendation. For patients with HF with preserved ejection fraction (HFpEF), a Class I recommendation is provided generically for the control of blood pressure and heart rate, with no medication classes specified. Beta blockers, ACE inhibitors, and ARB’s receive a Class IIb, level of evidence C recommendation, suggesting that these agents may be beneficial, although definitive evidence is lacking.

Two large population-based studies evaluating the frequency of hospitalizations for HF in the U.S. between 1970 and 2000 found relatively stable hospitalization rates [18, 19]. In contrast, a recent study of more than 55 million Medicare beneficiaries hospitalized with HF between 1998 and 2008 showed a marked decrease in the hospitalization rate for HF over this period [20]. While the use of evidence-based therapies to treat HF may have been partially responsible for the observed decrease in HF-related hospitalizations, few data are available describing changing trends in the utilization of, and factors associated with, HF medications in a large, community-based population.

The primary objective of this study was to describe decade-long trends (1995 - 2004) in the prescribing of in-patient and out-patient medications used to treat patients hospitalized with acute heart failure (ADHF). A secondary objective was to examine factors associated with the prescribing of recommended cardiac medications.

## Methods

The data for this study were derived from the Worcester Heart Failure Study, a population-based study of patients hospitalized with ADHF in the greater Worcester, MA, metropolitan area [21-23]. This study was approved by the Institutional Review Board at the University of Massachusetts Medical School.

The study sample consisted of greater Worcester residents hospitalized for possible ADHF during the 4 study years of 1995, 2000, 2002, and 2004. These years were selected to coincide with population census estimates and based on the availability of grant funding. Trained physicians and nurses performed a standardized review of the medical records of greater Worcester residents hospitalized at 11 medical centers in central Massachusetts with International Classification of Disease (ICD)-9 codes consistent with the presence of possible HF. A discharge diagnosis of HF (ICD-9 code 428) was the principal diagnostic category reviewed. In addition, the medical records of patients with additional discharge diagnoses were reviewed to identify patients who may have had new onset HF. The diagnosis of HF was confirmed based on use of the Framingham Study criteria [24]. We included in our study population patients with an initial (incident) episode of ADHF as well as those with previously diagnosed HF.

Data on patient demographics, medical history, clinical characteristics, presenting symptoms, laboratory and test results, as well as prescribing of cardiac medications on admission, during hospitalization, and at the time of hospital discharge were collected. Medications of interest included those shown to improve survival in patients with HFrEF (beta blockers, ACE inhibitors, ARBs, and aldosterone antagonists), therapies designed to provide symptomatic relief (diuretics, digoxin, calcium channel blockers, and nitrates), and therapies shown to be beneficial in patients with HF due to coronary artery disease (aspirin and statins). We did not collect information on post-discharge medication use.

### Data analysis

Chi square tests for trend were utilized to examine the significance of changes in the prescribing of selected cardiac medications between 1995 and 2004. Chi-square tests were also used to evaluate differences in discrete variables, while t-tests were used to evaluate differences in continuous variables, between respective comparison groups. We used logistic multivariable regression modeling to evaluate the association between various patient characteristics and the lack of treatment with the medications of interest. These variables were selected on the basis of the results of prior studies and on our observed univariate associations with the use of the different medications examined. We also used logistic regression modeling to examine changing trends in hospital discharge medication use, relative to the referent year of 1995, while simultaneously controlling for age, sex, race, history of several cardiovascular and noncardiovascular comorbidities, acute presenting symptoms, length of hospital stay, and several physiologic parameters (for example, estimated GFR, blood pressure, serum glucose levels). Analysis of pre-hospital medications was only examined in patients with previously diagnosed HF (incident patients excluded), while the analysis of medications at the time of hospital discharge was examined in hospital survivors only. We did not examine the association between the prescribing of selected medications and either hospital or post-discharge death rates due to the potential confounding for medication indication and due to the nonrandomized nature of this descriptive study. Ejection fraction (EF) findings were assessed in approximately one-third of study patients during their index hospitalization.

## Results

The study population consisted of 9,748 adult residents of the Worcester metropolitan area hospitalized with ADHF. The mean age of this population was 76 years, 94% were Caucasian, 44% were male, and 71% had been previously diagnosed with HF. Many patients had important comorbidities and most presented with dyspnea and edema (Table 1).

**Table 1:** Demographic and Clinical Characteristics of Patients Hospitalized With Acute Heart Failure

	Total Population	1995 Cohort	2004 Cohort
	(n = 9,748)	(n = 1,949)	(n = 2,469)
Age (mean, years)	76.2	75.7	76.2
Age group (years) (%)			
< 65	15.6	14.3	16.7
65 - 74	21.5	26.9	20.8
75 - 84	37.1	37.5	34.1
≥ 85	25.7	21.4	28.4
Male	43.9	42.9	46.9
Caucasian	93.8	96.8	92.3
Incident event	29.0	26.1	36.4
Length of hospital stay (days)	6.1	7.4	6.1
Physiologic Factors			
Cholesterol (mg/dL)	163.1	178.9	149.9
Systolic blood pressure (mmHg)	142.9	146.3	140.6
Diastolic blood pressure (mmHg)	74.7	79.1	72.5
eGFR (mL/min/1.73 m2)	52.7	54.1	51.8
Glucose (mg/dL)	160.6	169.5	152.9
Medical History (%)			
Anemia	24.6	21.9	26.7
Coronary heart disease	56.0	57.0	55.7
Chronic lung disease	35.9	35.5	35.7
Diabetes	39.0	39.7	39.3
Hypertension	68.7	62.3	72.8
Peripheral vascular disease	19.7	20.2	21.0
Renal failure	25.9	21.5	31.4
Stroke	13.2	14.3	11.5
Presenting Symptoms (%)			
Angina/chest pain	31.2	31.2	29.9
Dyspnea/shortness of breath	93.4	96.5	92.2
Edema/swelling	70.3	63.8	72.7
Nausea/vomiting	16.0	14.2	17.1
Orthopnea	35.4	29.2	38.4
Weakness	25.1	24.9	23.8

Data are given as percentages or means unless otherwise noted.

Patients hospitalized with ADHF in 2004 as compared to the initial study year of 1995 were more likely to be older, male, and non-Caucasian, to have a higher body mass index, lower blood pressure and serum cholesterol levels, and to present with an initial episode of ADHF (Table 1). They were also more likely to have a history of hypertension or renal insufficiency. Among the 3,611 patients who had EF information available, the average and median EF values were 45% and 48%, respectively; among these patients, 1,786 (49%) had EF findings of 50% and higher.

### Trends in medication use

The use of beta blockers, angiotensin pathway inhibitors (ACE inhibitors and ARBs), statins, and aspirin during hospitalization for ADHF increased markedly during the study period (Table 2). Between 1995 and 2004, the use of beta blockers increased nearly three -fold, the use of angiotensin pathway inhibitors increased from 50.4% to 60.7%, and the use of statins increased more than seven fold. Similar trends were noted for the prescribing of chronic pre-hospital (out-patient) as well as discharge medications. The use of diuretics was widespread and relatively constant during the years under study (Table 2). In contrast, the in-patient and out-patient use of digoxin, nitrates, and calcium channel blockers decreased markedly during the years under study.

**Table 2:** Trends in Selected Medication Use in all Patients Hospitalized With Acute Heart Failure

	ACE-I/ ARBs	Aldosterone Antagonists	Aspirin	Beta Blockers	Calcium Channel Blockers	Digoxin	Diuretics	Nitrates	Statins
Pre-Hospital (%)									
1995	43.7	na	28.0	20.1	35.5	49.9	82.9	46.4	6.0
2000	48.2	na	39.6	44.9	27.2	41.1	81.1	38	20.0
2002	55.3	8.0	43.4	55.3	24.6	37.0	84.0	31.6	33.4
2004	55.2	9.4	48.8	67.9	24.6	29.7	83.4	29.5	46.3
In-Hospital (%)									
1995	50.4	na	38.9	26.2	40.2	55.0	97.9	67.9	5.8
2000	55.9	na	56.4	51.8	33.0	47.2	97.7	62.5	21.0
2002	58.6	9.3	59.0	62.5	29.8	41.2	96.6	53.0	32.8
2004	60.7	11.3	63.1	73.7	29.7	33.3	96.4	47.1	44.9
Discharge (%)									
1995	46.5	na	35.0	22.6	32.8	50.9	82.5	45.6	5.4
2000	51.9	na	47.6	46.8	26.2	42.6	82.3	35.6	20.6
2002	54.2	7.7	48.6	56.5	22.9	36.7	80.1	27.4	30.9
2004	54.8	9.1	51.0	67.5	22.5	29.1	81.7	24.5	43.4

Pre-hospital data excludes incident cases of acute HF; Hospital discharge data excludes hospital decedents; ACE-I/ARBs = angiotensin converting enzyme inhibitors or angiotensin receptor blockers.

Given the differences in recommended therapy for HFrEF compared to HFpEF, we examined trends treatment practices in these 2 groups (Table 3 and Table 4). In the 1,330 patients with HFrEF (HF ≤ 40%), there were marked increases in the administration of beta blockers, angiotensin pathway inhibitors, aspirin, and statins over time, in accordance with guidelines (Table 3). In the 1,786 patients with HFpEF (EF ≥ 50%), there were substantial increases in the prescribing of beta blockers, angiotensin pathway inhibitors, aspirin and statins (Table 4), though of a lesser magnitude overall than in patients with HFrEF.

**Table 3:** Trends in Medication Use in Patients With HFrEF

	ACE-I/ ARBs	Aldosterone Antagonists	Aspirin	Beta Blockers	Calcium Channel Blockers	Digoxin	Diuretics	Nitrates	Statins
Pre-Hospital (%)									
1995	44.4	N/A	28.0	19.6	34.9	46.6	76.7	43.4	6.4
2000	52.6	N/A	39.1	44.7	21.7	44.3	78.0	40.1	28.4
2002	64.6	5.7	43.4	57.1	16.0	42.9	82.0	29.4	36.6
2004	59.0	9.5	50.2	70.0	20.2	32.6	77.3	30.0	52.0
In-Hospital (%)									
1995	67.3	N/A	52.5	31.2	38.6	64.8	97.8	72.8	5.9
2000	74.3	N/A	63.9	57.1	28.4	65.3	98.6	67.4	27.8
2002	79.2	10.8	68.7	72.4	23.8	57.7	96.7	53.5	37.3
2004	73.0	16.4	73.0	82.4	24.3	46.7	96.6	52.7	49.5
Discharge (%)									
1995	59.6	N/A	42.4	21.9	25.2	58.9	80.8	45.7	4.6
2000	69.8	N/A	53.4	48.5	18.3	60.2	87.0	34.4	26.8
2002	72.3	9.5	55.0	66.1	15.0	52.2	83.4	26.1	33.5
2004	66.0	14.7	58.2	74.4	14.0	40.5	79.5	28.7	46.8

Pre-hospital data excludes incident cases of acute HF; Hospital discharge data excludes hospital decedents; ACE-I/ARBs = angiotensin converting enzyme inhibitors or angiotensin receptor blockers

**Table 4:** Trends in Medication Use in Patients With HFpEF

	ACE-I/ ARBs	Aldosterone Antagonists	Aspirin	Beta Blockers	Calcium Channel Blockers	Digoxin	Diuretics	Nitrates	Statins
Pre-Hospital (%)									
1995	34.7	N/A	22.8	27.7	49.5	33.7	78.2	39.6	8.9
2000	38.2	N/A	40.8	41.2	39.0	29.2	78.7	27.0	19.5
2002	46.3	6.4	37.2	57.9	32.6	25.6	83.2	25.9	32.9
2004	52.3	6.6	45.6	62.0	33.8	22.3	81.5	21.6	40.8
In-Hospital (%)									
1995	45.0	N/A	45.0	29.8	56.7	45.0	98.8	68.4	5.3
2000	49.1	N/A	57.6	51.6	45.1	35.3	97.8	59.8	20.3
2002	48.2	7.5	59.4	62.4	37.6	29.6	97.6	51.1	28.1
2004	56.8	5.6	61.5	70.5	40.6	22.7	97.7	42.4	40.3
Discharge (%)									
1995	36.7	N/A	38.0	25.3	43.0	39.9	76.0	39.2	5.1
2000	43.5	N/A	47.8	44.9	35.2	27.2	74.0	25.3	20.6
2002	41.7	6.4	46.2	54.9	28.1	23.7	76.9	22.0	25.9
2004	46.6	3.7	46.2	62.8	31.8	15.8	77.1	16.6	38.4

Pre-hospital data excludes incident cases of acute HF; Hospital discharge data excludes hospital decedents; ACE-I/ARBs = angiotensin converting enzyme inhibitors or angiotensin receptor blockers.

Given changing patient demographic and clinical characteristics during the years under study, we examined changing trends in hospital discharge medication use while simultaneously controlling for a variety of demographic and clinical characteristics (Table 5). Compared to the referent year of 1995, patients hospitalized with ADHF in 2004 were significantly more likely to have been prescribed ACE inhibitors/ARBs, aspirin, beta blockers, and lipid lowering medication, and were significantly less likely to have been treated with calcium channel blockers, digoxin, and nitrates, at the time of hospital discharge. Relatively similar trends were observed in patients with HFpEF and HFrEF with these trends somewhat more pronounced in patients with HFrEF (Table 5).

**Table 5:** Changing Trends in Hospital Discharge Medication Use in Patients With Acute Heart Failure

	Total Population			Preserved Ejection Fraction Findings			Reduced Ejection Fraction Findings		
	
Medication	2000	2002	2004	2000	2002	2004	2000	2002	2004
	(n = 2,587)	(n = 2,743)	(n = 2,469)	(n = 448)	(n = 591)	(n = 576)	(n = 490)	(n = 518)	(n = 493)

	O.R.	O.R.	O.R.	O.R.	O.R.	O.R.	O.R.	O.R.	O.R.
ACE inhibitors/ARB’s	1.26 (1.10, 1.43)*	1.42 (1.25, 1.62)*	1.54 (1.35, 1.76)*	1.34 (0.90, 2.00)*	1.27 (0.86, 1.88)*	1.63 (1.10, 2.42)*	1.65 (1.19, 2.28)*	1.93 (1.39, 2.69)*	1.49 (1.07, 2.07)*
Aspirin	1.81 (1.59, 2.07)	1.90 (1.66, 2.17)	2.13 (1.86, 2.45)	1.56 (1.04, 2.33)	1.50 (1.01, 2.22)	1.52 (1.02, 2.25)	1.75 (1.29, 2.39)	1.91 (1.39, 2.60)	2.33 (1.70, 3.20)
Beta blockers	3.51 (3.03, 4.06)	5.46 (4.71, 6.33)	8.92 (7.64, 10.40)	2.85 (1.84, 4.43)	4.52 (2.93, 6.96)	6.52 (4.20, 10.14)	4.15 (2.91, 5.91)	9.55 (6.62, 13.78)	14.25 (9.74, 20.83)
Calcium channel blockers	0.70 (0.61, 0.80)	0.54 (0.47, 0.63)	0.53 (0.45, 0.61)	0.69 (0.46, 1.03)	0.45 (0.30, 0.67)	0.53 (0.36, 0.80)	0.65 (0.44, 0.94)	0.46 (0.31, 0.68)	0.44 (0.29, 0.66)
Digoxin	0.71 (0.63, 0.81)	0.58 (0.51, 0.65)	0.42 (0.37, 0.49)	0.63 (0.42, 0.95)	0.53 (0.35, 0.79)	0.34 (0.22, 0.51)	0.98 (0.72, 1.34)	0.69 (0.51, 0.95)	0.47 (0.34, 0.64)
Diuretics	0.87 (0.73, 1.03)	0.76 (0.64, 0.90)	0.97 (0.81, 1.16)	0.76 (0.48, 1.22)	0.93 (0.59, 1.48)	1.03 (0.64, 1.64)	1.41 (0.93, 2.14)	1.00 (0.67, 1.50)	0.82 (0.55, 1.21)
Lipid lowering medications	5.37 (4.24, 6.80)	10.02 (7.94, 12.64)	18.52 (14.65, 23.41)	6.17 (2.81, 13.53)	8.40 (3.88, 18.21)	17.01 (7.83, 36.96)	8.26 (4.56, 14.95)	12.54 (6.94, 22.66)	24.61 (13.59, 44.57)
Nitrates	0.62 (0.54, 0.71)	0.40 (0.35, 0.47)	0.36 (0.31, 0.42)	0.42 (0.27, 0.66)	0.36 (0.23, 0.55)	0.23 (0.15, 0.36)	0.57 (0.41, 0.80)	0.36 (0.26, 0.51)	0.41 (0.29, 0.59)

*95% Confidence Intervals.

Data on the use of aldosterone antagonists were only available for patients hospitalized in 2002 and 2004; approximately 10% of patients with HF were treated with aldosterone antagonists during these 2 years. Among patients with HFpEF, 6.5% of patients were treated with these agents in 2002 and 2004 whereas 13.6% of patients with HFrEF were treated with aldosterone antagonists during these years.

### Contraindications to treatment

To better understand the possible reasons for the lack of use of recommended medications in certain patients hospitalized with ADHF, we reviewed the charts of all study patients for contraindications to these agents. The frequency of listed allergy/intolerance to all study medications increased slightly from 1995 to 2004, but was quite low in all cases. Rates of allergy/intolerance to medications in the most recent study year (2004) were as follows: ACE inhibitors/ARB’s 3.3%, aspirin 4.0%, aldosterone antagonists 0.1%, beta blockers 0.8%, and lipid-lowering medications 0.9%.

### Patient characteristics and receipt of selected cardiac medications

Women, the oldest patients (≥ 85 years of age), patients experiencing HF for the first time, patients with higher ejection fractions, patients with a history of anemia or chronic lung disease, and patients presenting with generalized weakness were the most likely to be discharged without either a beta blocker or an angiotensin pathway inhibitor. In 1995, 60.3% of patients were discharged on medications from at least 1 of the 2 recommended drug classes for the management of ADHF and 8.8% were discharged on medications from both drug classes; these percentages were 81.9% and 40.4%, respectively, in 2004 (P < 0.001).

After controlling for a variety of demographic and clinical factors, patients hospitalized in the initial study year of 1995, women, patients with a first hospitalization for ADHF, those with impaired renal function, a history of anemia or COPD, and those who presented with edema were significantly less likely to have received effective HF therapies at the time of hospital discharge than respective comparison groups. While relatively similar factors were associated with the failure to be treated with effective HF therapies in our initial (1995) and most recent (2004) study years, there were appreciably fewer demographic and clinical factors associated with the failure to receive these therapies in 2004.

## Discussion

The results of this community-wide study of patients hospitalized with ADHF demonstrate a marked increase in the use of beta blockers and angiotensin pathway inhibitors, in accordance with ACC/AHA Guidelines, and a decrease in the use of therapies that have not been shown to reduce patient mortality. Older patients, women, patients with a new presentation of HF, patients with non-cardiac co-morbidities, and patients presenting with generalized symptoms were the least likely to be treated with effective HF medications. Although fewer than 1 in every 5 patients with HF was discharged without a beta blocker or angiotensin pathway inhibitor in 2004, only 2 out of every 5 patients with ADHF were discharged on both of these recommended medication classes. These results suggest that there are opportunities for further improvement in the medical management of patients with ADHF. Importantly, the increased use of recommended medications documented in this study is coincident with a marked decrease in the HF hospitalization rate that has been observed nationally [20].

### Trends in HF medication prescribing practices

Several studies have documented a change in medication prescribing patterns for patients with HF following the initial publication of ACC/AHA HF guidelines in 1995 and following more recent updates. The Cardiovascular Health Study evaluated the use of discharge medications for elderly persons with incident HF between 1989 and 2000 in 4 large U.S. communities [25]. In this study, there was an average increase in beta blocker use of 2.4% annually between 1989 and 2000.

A study based on data from the Acute Decompensated Heart Failure National Registry (ADHERE) examined quarterly trends in treatment practices in 159,168 HF hospitalizations from 2002 to 2004 [26]. Over this period, beta blocker use increased by approximately 20%. The use of angiotensin pathway inhibitors and diuretics was relatively unchanged, the use of aldosterone antagonists increased slightly, and the use of digoxin decreased significantly. In 2004, approximately 62% of HF cases were treated with an angiotensin pathway inhibitor prior to hospitalization, compared with 55% in our study, while 69% were treated with a beta blocker, compared with 68% in our study.

The results of the present study confirm the findings of earlier investigations, which showed improved adherence to ACC/AHA guidelines. Our study extends prior results by demonstrating continuous improvement in the prescribing of recommended HF therapies between 1995 and 2004, following initial issuance of these guidelines in 1995. The present study also extends prior work by evaluating the combination use of effective HF therapies, as well as the effect of medication contraindications, demographic, and clinical factors on prescribing practices.

The observed increases in the use of effective cardiac therapies for HF is notable in the context of the significant decrease in the hospitalization rate for HF observed nationally in more than 55 million Medicare beneficiaries hospitalized with HF between 1998 and 2008 [20]. The decreased rate of HF hospitalizations over this period stands in contrast with the results of prior studies, such as the Framingham Heart Study (1970 - 1999) [18], and Olmsted County, MN, study (1979 - 2000) [19], which covered periods prior to the issuance of the ACC/AHA HF guidelines, and demonstrated stable rates of HF-related hospitalizations. While we cannot directly assess the contribution of improved medical therapy to the recent reduction in hospitalization rate for HF, it has no doubt played an important role.

### Patient factors and receipt of cardiac therapies

In the limited studies that have examined patient characteristics associated with the use of different HF therapies, the underuse of beneficial cardiac therapies has been previously related to older patient age, impaired renal function, and preserved left ventricular systolic function. In the current study, patients of advanced age, women, those with selected comorbidities, and those with a first episode of ADHF were least likely to receive effective treatment modalities.

Although avoidance of beta-blockers in patients with asthma or COPD may be prudent, a substantial proportion of these patients can tolerate careful titration of beta-blockers without respiratory compromise. Underutilization of effective therapies in patients with a first episode of HF may represent a missed opportunity to prevent recurrent events.

Our findings support the increasing enthusiasm by quality control agencies and hospitals for the development of comprehensive guidelines for the management of patients with acute and chronic HF. These guidelines strongly recommend the use of both pharmacologic and non-pharmacologic management, emphasize close patient follow-up, and stress patient education and involvement.

### Study strengths and limitations

The primary strengths of this study were the large, population-based sample of patients with independently validated ADHF, detailed information on out-patient, in-hospital, and discharge medications, and extensive information on patient demographic and clinical characteristics. Limitations of this study included a predominantly Caucasian population from a single region in central New England, inability to validate patient adherence to the medications examined, and lack of information regarding why certain patient subgroups may have been undertreated with effective HF treatment regimens. The inability to more systematically examine the use of the therapies under study in relation to whether patients had preserved or reduced EF findings, since only one third of patients had EF data available, is an acknowledged limitation of this investigation.

### Conclusions

In this community-wide study, the use of recommended therapies for HF increased markedly following issuance of the ACC/AHA HF guidelines. While progress in the management of these high risk patients has been substantial, and recent data shows an encouraging decrease in the rate of hospitalization for HF, there remains considerable opportunity for more optimal medical treatment of patients with HF.
